# Clinical and radiographic diagnostic study of strontium ranelate andmetal-substituted hydroxyapatite bone graft materials in diabetesmellitus with chronic periodontitis

**DOI:** 10.34172/japid.2020.015

**Published:** 2020-12-09

**Authors:** Omar Khashaba, Atef Alasfar, Enas Ahmed Elgendy, Bassant Mowafey

**Affiliations:** ^1^Professor of Oral Medicine, Periodontology, Oral Diagnosis, and Oral radiology Department, Faculty of Oral and Dental Medicine, Mansuora University, Egypt; ^2^M.S.C, Faculty of Oral and Dental Medicine, Mansuora University, Egypt; ^3^Professor of Oral Medicine, Periodontology, and Oral Diagnosis Department, Faculty of Oral and Dental Medicine, Kafr El-Sheikh University, Egypt; ^4^Lecturer of Oral Diagnosis and Oral Radiology, Faculty of Oral and Dental Medicine, Mansuora University, Egypt

**Keywords:** Chronic periodontitis, Cone-beam computed tomography, Diabetes mellitus, Metal-substituted hydroxyapatite, Periodontal flap, Strontium ranelate

## Abstract

**Background:**

The present study aimed to assess the clinical and radiographic effect of strontium ranelate and metal-substituted hydroxyapatite as bone graft materials on treating chronic periodontitis among diabetes mellitus patients.

**Methods:**

A randomized split-mouth study was conducted on 20 sites in 10 controlled type II diabetic patients suffering from chronic periodontitis. After phase I therapy, the sites were randomly allocated by a computer-generated table into two groups. Group 1: A mucoperiosteal flap was elevated in 10 sites, followed by the placement of strontium ranelate mixed with Gengigel. Group 2: A mucoperiosteal flap was elevated in 10 opposite sites, followed by the placement of metal-substituted hydroxyapatite mixed with Gengigel. Clinical parameters were assessed at baseline and 3- and 6-month intervals. Cone-beam computed tomography (CBCT) was used at baseline and after six months to assess bone gain.

**Results:**

The two treatment modalities resulted in a statistically significant reduction in clinical parameters at the 3- and 6-month intervals compared to the mean baseline value. Intergroup comparison showed a significant reduction in probing pocket depth and clinical attachment loss in group 1 compared to group 2. Comparison of the two sides by CBCT showed a significant increase in the alveolar bone height in the SR group than the metal-substituted hydroxyapatite group.

**Conclusion:**

Clinical and radiographic results showed a significant improvement in the two groups and provided evidence that strontium ranelate is promising in treating periodontal diseases.

## Introduction

There is a clear relationship between the degree of hyperglycemia and the severity of periodontitis.^1^ The possible mechanisms by which DM might affect the periodontium are increased counts of subgingival pathogenic bacteria such as Capnocytophaga, anaerobic vibrios, Actinomyces species, Porphyromonas gingivalis, Prevotella intermedia, and Aggregatibacter actinomycetemcomitans. Neutrophil impairment leads to increased susceptibility to periodontitis in people with diabetes. Hyperglycemia induces decreased chemotaxis, phagocytosis, and intracellular bacterial activity in diabetics. Collagen from diabetes patients has been reported to be more insoluble and resistant to digestion, directly impairing degradation, and remodeling. The basement membrane protein undergoes non-enzymatic glycosylation when subjected to hyperglycemic conditions. The gingival capillaries of diabetic patients have greater basement membrane thickness.^2^

Alveolar bone loss is a hallmark of periodontitis progression, and its prevention is a crucial clinical challenge in periodontal disease treatment. Periodontal treatment aims to eliminate biofilms, remove microbial deposits from the root surface, decrease tissue destruction, and regenerate lost tissues.^3^

Bone grafts are frequently used in oral surgeries to facilitate or promote bone regeneration, provide mechanical support for the membrane, fill bone defects, increase bone augmentation, and stabilize blood clots.Many bone graft materials are available to resolve bone defects, including autografts, allografts as freeze-dried bone from human donors, xenografts from animals or plants, and alloplasts, which are synthetic bone substitutes as polymers, bioactive glass, hydroxyapatite, and beta-tricalcium phosphate. All these materials are not perfect substitutes for bones and have some advantages and disadvantages.^4^

A new material, referred to as strontium ranelate (SR), is used to replace bone loss. This material is composed of two atoms of stable strontium combined with ranelic acid, which serves as a carrier. Strontium ranelate, which is used for osteoporosis treatment, is the first drug to exhibit a dual action on bone metabolism. It combines the antiresorptive effects by inhibiting osteoclastic differentiation and activity with an anabolic effect on bone formation by stimulating osteoblastic differentiation, activity, and collagen synthesis.^5^

Hydroxyapatite (HA) is a commonly used bone graft material and is a well-documented biomaterial known for its osteoconductivity.^6^ Iron oxide nanoparticles are currently used for these applications; however, there are concerns over acute toxicity. Since HA is biocompatible and biodegradable for dental and hard tissue grafts, magnetic HA application will mitigate these concerns.^7^ Besides, iron oxide nanoparticles can play a positive role in enhancing the magnetic properties of the product. These particles have to be integrated into the structure of hydroxyapatite particles.^8^

Conventional radiographs generate 2D images in which the roots are superimposed on the bone; thus, bone changes, such as furcation involvement and buccal and lingual bone changes, are difficult to observe.^9^ The cone-beam computed tomography (CBCT) has been used to visualize the interproximal defects and buccal and lingual bone defects for diagnosing dehiscence and fenestration defects for evaluating diagnostic and treatment outcomes of periodontitis, evaluating postsurgical results of regenerative periodontal therapy, measuring alveolar bone density, and assessing the healing process after graft placement in periodontology.^10^

This study aimed to compare SR and metal-substituted hydroxyapatite (MSHA) bone graft materials for treating chronic periodontitis in patients with diabetes mellitus.

## Methods

### Patient Selection

This study included 20 sites in controlled type II diabetic patients with severe chronic periodontitis selected from the Periodontology Clinic, Faculty of Dentistry, Mansoura University. The patients’ ages ranged from 35 to 55 years, their consent was taken, and all the procedures were explained before treatment.

All the procedures followed the ethical standards of the Committee on Human Experimentation (institutional and national) and the Helsinki Declaration, as revised in 2013. The study protocol was approved by the relevant Research Ethics Committee.

### Inclusion Criteria

The selected patients fulfilled the following criteria:

- At least 20 teeth remaining in the oral cavity
- Pocket probing depth (PPD) ≥5 mm
- Clinical attachment level (CAL) ≥5 mm
- Vertical bone loss on radiographic examination
- Patients who are able to maintain good oral hygiene
- Glycosylated hemoglobin (HbA1C) in controlled diabetics ≤7%

### Exclusion Criteria

- History of systemic diseases other than type II diabetes
Smoking
- Pregnant or lactating women
- Previous treatment of periodontal diseases in the last six months

### Materials

#### 1. Osteostatin (Strontium Ranelate)

Bone graft material for dental use was manufactured in the Arab Republic of Egypt (ARE) and approved by the Solicitors Regulation Authority (SRA). It is composed of two atoms of stable strontium combined with ranelic acid, which acts as a carrier.

#### 2. Metal Substituted Hydroxyapatite

Pure HA nanoparticles doped with Mn^2+^ and Fe^3+^ ions were synthesized using the wet chemical method (WCM). The samples were formed by different spectroscopic techniques in the Department of Physics, Faculty of Science at Mansoura University.

#### 3. Gengigel

Gengigel was manufactured by Ricerfarma in European Union. It is composed of 0.2% hyaluronic acid and 7.5% xylitol (extracted from birch and beech trees).

### The Clinical Study Design

#### 1. Phase I Therapy

All the patients were subjected to full-mouth scaling and root planing (SRP) using hand instruments and ultrasonic and proper oral hygiene instructions. In addition, an occlusal adjustment was performed if signs of trauma were evident. One month after phase I therapy, all the patients were evaluated to ensure the surgical procedure’s local fitness. All the patients performed a glycosylated hemoglobin analysis before periodontal surgery.

#### 2. Patient Groups

After phase I therapy, a randomized split-mouth study was used on the selected patients to receive one of the two proposed treatments on one side and the other treatment on the contralateral side.

This randomization was achieved by a computer-generated table.

**First, proposed treatment (group 1):** It was applied in 10 randomized sides and included a surgical flap with a mix of 1 g of SR with Gengigel.

**Second, proposed treatment (group 2):** Itwas applied in 10 contralateral sides and included a surgical flap with a mix of 1 g of MSHA bone graft materials with Gengigel.

#### 3. Clinical Assessment

A proper case history was obtained from each patient considering the history of the present chief complaint, onset and duration of the patients’ periodontal manifestations, and any past dental treatment. Each patient was thoroughly examined clinically to assess the gingival tissue condition in terms of color, size, texture, and contour. Clinical evaluation was performed using the following clinical parameters:

- Plaque index (PI): at the baseline and after 1, 3, and 6 months^11^
- Gingival index (GI): at the baseline and after 1, 3, and 6 months^12^
- Probing pocket depth (PPD): at the baseline, 3, and 6 months only^13^
- Clinical attachment level (CAL): at the baseline, 3, and 6 months, only^13^

#### 4. Radiographic Examination

The bone level of teeth indicated in the surgery was measured by cone-beam images (i-CAT Next Generation Machine, Imaging Sciences International, Hatfield, PA, USA).

Cone-beam CT was taken at baseline and six months after surgery for measuring changes in the bone level of the indicated bone defect site.

- Preoperative bone defect (at baseline) was measured as the distance from the cementoenamel junction (CEJ) to the bone defect base.
- Postoperative bone defect (after six months) was measured as the distance from the CEJ to the base of the bone formation.
- Postoperative bone fill (bone gain after six months) was deducted by subtracting the postoperative bone defect from the preoperative bone defect.

#### 5. The Surgical Phase

**Figure 1:**
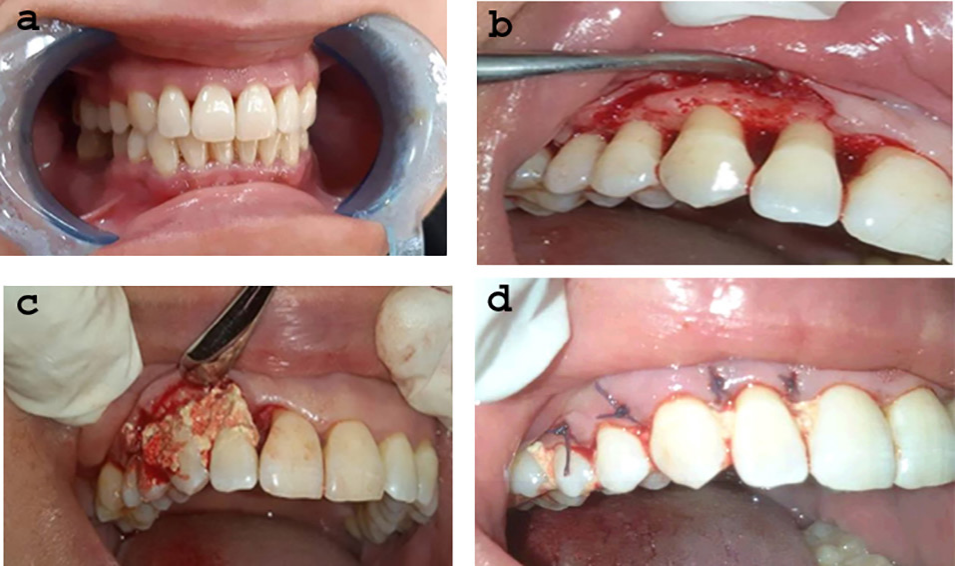
a, Photograph showing the case after phase I therapy, b, Full thickness flap reflected to expose the intrabony defect with care to preserve the interdental papilla, c, The mix of strontium ranelate and gengigel placed into defect site until filled, d, Suturing with 0.3 mm of non resropable suture.

**Figure 2:**
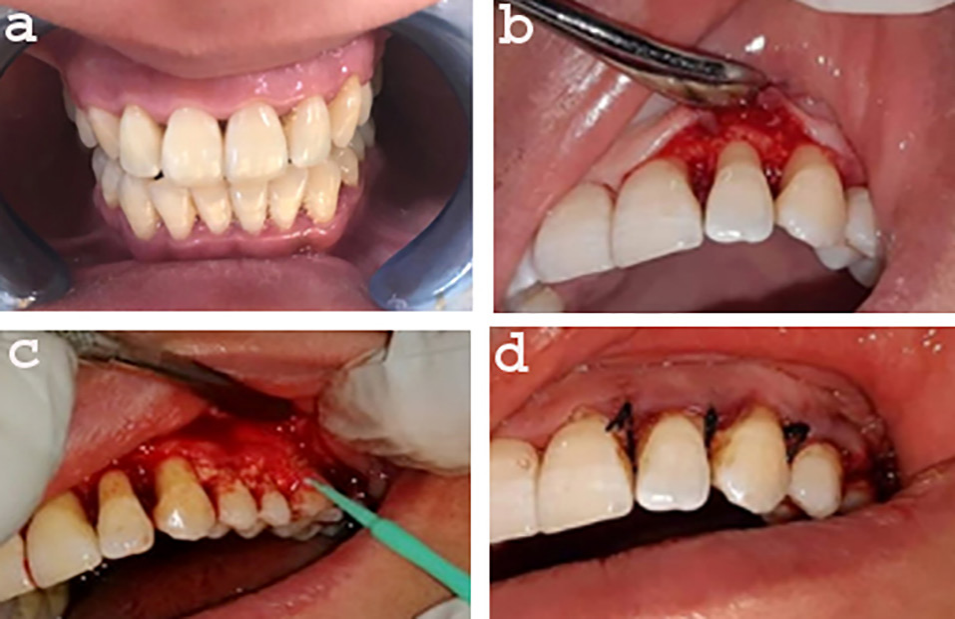
a, Photograph showing the case after phase I therapy, b, Full thickness flap reflected to expose the intrabony defect with care to preserve the interdental papilla, c, The mix of metal substituted hydroxyapatite and gengigel placed into defect site until filled, d, Suturing with 0.3 mm of non resropable suture.

After phase I therapy, andadequate local anesthesia, buccal and lingual intrasulcular incisions were made using Bard-Parker blade #15, and a full-thickness flap was reflected to expose the intrabony defects, with care to preserve the interdental papilla. After debridement of the osseous defects, the root surfaces were thoroughly scaled and planed with Gracey curettes.Then according to the selected treatment modality, either SR (Osteostatine) or MSHA was mixed with 2–4 drops of hyaluronic acid (Gengigel) to achieve a putty consistency that was carried by a spoon-like instrument into the defect site until filled, and then condensed gently with a sterile smooth amalgam condenser. Suturing was carried out with 0.3 nonresorbable sutures (Figures 1 and 2).

#### 6. Postoperative Care

1. Amoxicillin (500 mg) was prescribed twice daily for seven days.

2. Non-steroidal anti-inflammatory drug (NSAID) was prescribed every eight hours for two days to control postoperative discomfort (Ibrofen, 600 mg).

3. A soft diet and a mouth rinse with 0.12% chlorhexidine solution were recommended twice daily for 10 days.

4. The patient was instructed to avoid hard and spicy food post-surgically.

5. Two weeks after the operation, the sutures were removed, and all the patients were instructed to use a soft toothbrush for plaque control.

6. All the patients were recalled for professional prophylactic and plaque control procedures once every two weeks during the first month and once a month for six months.

### Statistical Analysis

All the data were collected, tabulated, and statistically analyzed using SPSS 21. The difference between the two groups was analyzed using the independent sample (Student) t-test, and intergroup comparison was performed using a paired t-test. P-values<0.05 were considered statistically significant.

## Results

**Figure 3:**
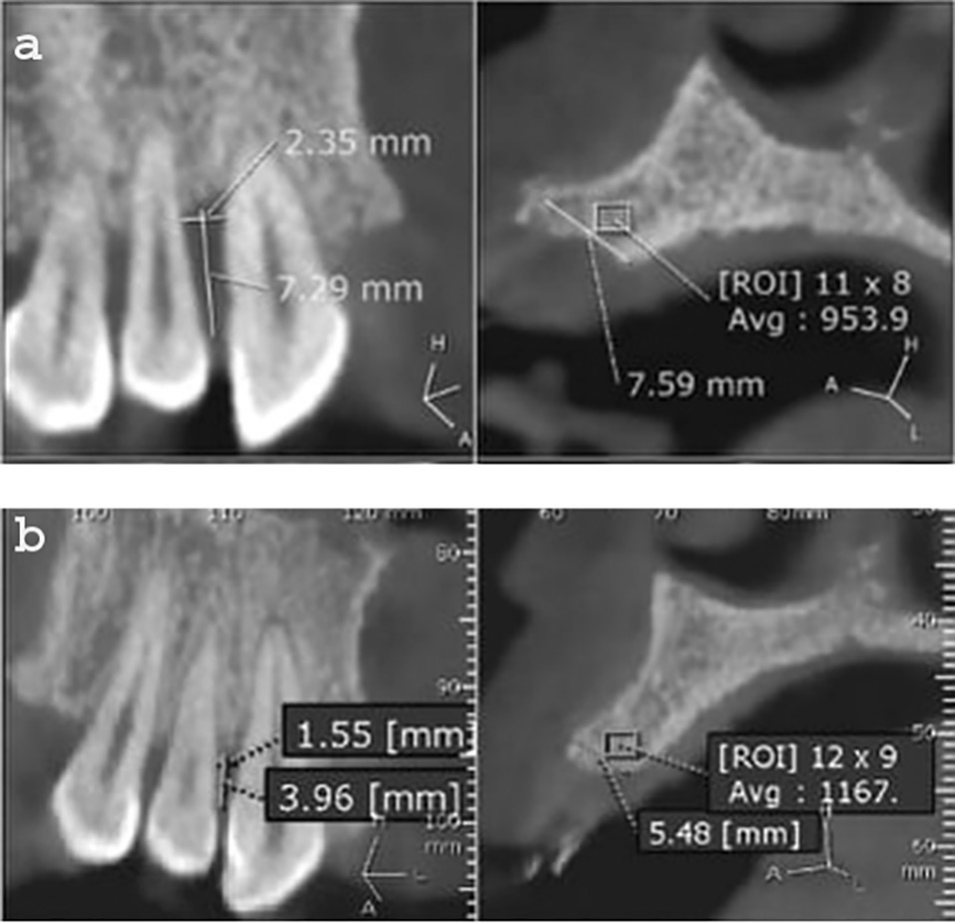
a, A cone beam computed tomography for measurement of the bone level of the right site defect of the case at baseline, b, A cone beam computed tomography for measurement of the bone level of the right site defect treated by strontium ranelate after 6 months.

**Figure 4:**
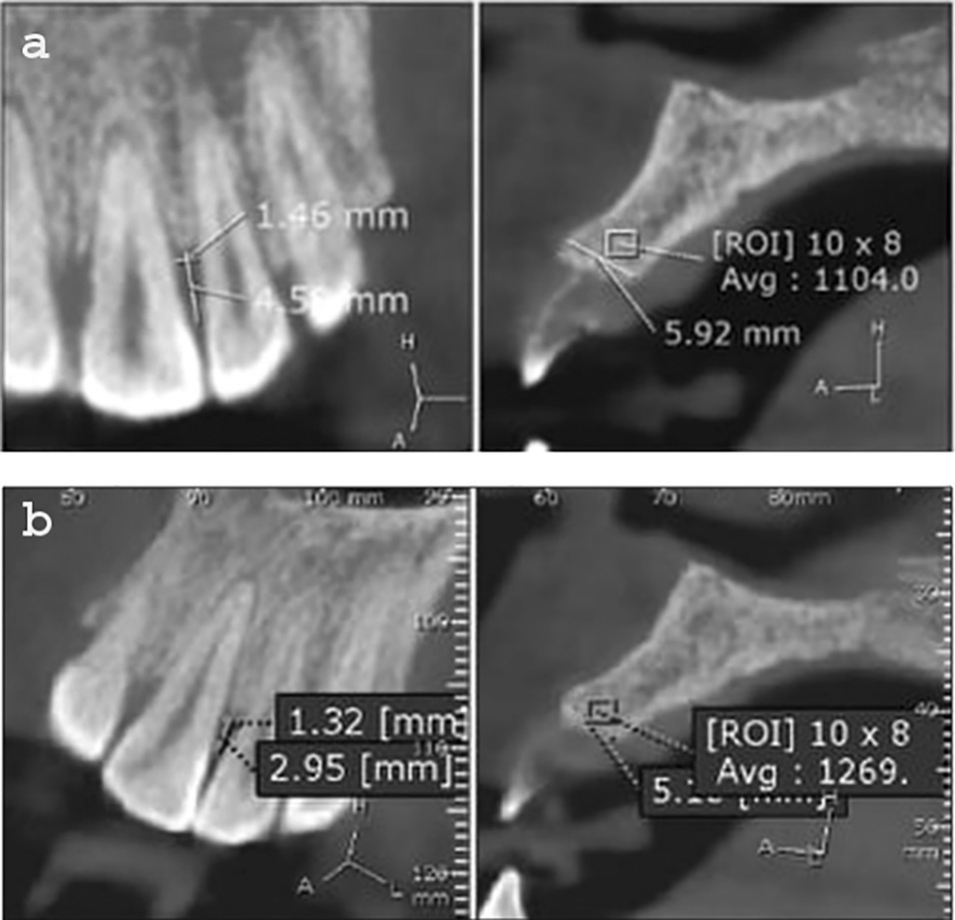
a, A cone beam computed tomography for measurement of the bone level of the left site defect of the case at baseline, b, A cone beam computed tomography for measurement of the bone level of the left site defect treated by metal substituted Hydroxyapatite after 6 months.

The two treatment modalities showed a statistically significant reduction in PPD and CAL at three and six months compared to the mean baseline value. Intergroup comparison showed that group 1 had significantly less PPD and CAL compared to group 2. Comparing the two sides by CBCT showed an increase of nearly 3.4 mm in the height of the alveolar bone on the group 1 (SR) side while the opposite side in group 2 (MSHA) showed a nearly 1.4 mm increase in the height of the alveolar bone (Tables 1 and 2) (Figures 3 and 4).

**Table 1:** Mean values of plaque index (PI), gingival index (GI), probing pocket depth (PPD) in mm, clinical attachment level (CAL) in mm, among the study groups at baseline, 3 and 6 months and after treatment

**Variable**	**Groups/Time**	**Group I** **n = 10** **mean ± SD**	**Group II** **n = 10** **mean ± SD**	**P-value, independent samples t-test**
**PI**	Baseline (1)	2.42±0.42	2.55±0.43	0.647 ^ns^ 0.526
	1 month (2)	0.77±0.27	0.87±0.35	0.700^ns^ 0.493
	3 months (3)	0.65±0.33	0.82±0.28	1.244^ns^ 0.229
	6 months (4)	0.67±0.23	0.85±0.31	1.400^ns^ 0.179
**Paired samples t-test**		0.0001 1vs2&3&4***	0.0001 1vs2&3&4***	
**GI**	Baseline (1)	2.30±0.30	2.28±0.20	0.129^ns^ 0.899
	1 month (2)	0.60±0.17	0.47±0.27	1.213^ns^ 0.241
	3 months (3)	0.67±0.12	0.52±0.24	1.716^ns^ 0.103
	6 months (4)	0.67±0.20	0.80±0.22	1.282^ns^ 0.216
**Paired samples t-test**		0.0001 1vs2&3&4***	0.0001 1vs2&3&4***	
**PPD**	Baseline (1)	5.80±0.63	6.00±0.66	0.688^ns^ 0.500
	3 months (2)	2.65±0.47	3.40±0.65	2.923† 0.010
	6 months (3)	2.40±0.51	3.10±0.56	2.885† 0.010
**Paired samples t-test**		0.0001 1vs 2*** 1vs 3***	0.0001 1vs 2*** 1vs 3***	
**CAL**	Baseline (1)	5.05±0.49	5.50±0.52	1.964^ns^ 0.065
	3 months (2)	2.40±0.45	2.95±0.49	2.569 0.019†
	6 months (3)	2.10±0.31	2.80±0.42	4.200 0.001†
**Paired samples t-test**		0.0001 1vs2&3***	0.0001 1vs2&3***	

*Significant change over time within group compared with baseline value (Significance: *P<0.05. **P<0.01, ***P<0.001 ns = not significant). †Significant difference between the two groups at different follow-up periods (P<0.05). Group I = Patients treated with OFD with strontium ranelate bone graft material Group II = Patients treated with OFD with metal substituted hydroxyapatite bone graft material

**Table 2:** Mean values of vertical bone loss and bone gain (BG) among the study groups at baseline and 6 months post-operatively

**The study groups**	**Group 1** **(n=10)**	**Group 2** **(n=10)**	**Independent samples t-test** **P value**
**Baseline**	4.77±1.32	4.25±1.27	0.892^ns^ 0.384
**6 months**	1.71±0.43	2.33±0.60	2.067 0.018†
**Bone gain**	3.06±1.36	1.91±0.79	2.284 0.035†
**Paired samples t-test** **P**	7.648*** 0.000	7.054*** 0.000	

*Significant change over time within group compared with baseline value (Significance: *P<0.05. **P<0.01, ***P<0.001 ns = not significant). †Significant difference between the two groups at different follow-up periods (P<0.05). Group 1 = Patients treated with OFD with strontium ranelate bone graft material Group 2 = Patients treated with OFD with metal substituted hydroxyapatite bone graft material

## Discussion

As periodontal treatment’s critical problem is the difficulty in restoring the alveolar bone, regenerative therapy has found its way to improve periodontal therapy. Among these regenerative materials is SR, which has been used for the replacement of bone loss.^5^ Also, magnetic hydroxyapatite nanoparticles enable their use in numerous therapeutic applications in the field of regeneration.^14^ Hyaluronic acid plays a multifunctional role in periodontal tissue healing, aiding in periodontal disease treatment. Topical hyaluronic acid can be useful as an adjunctive treatment in gingivitis, chronic periodontitis, and during the postoperative period for implant and sinus lift procedures to induce faster healing and reduce the patients’ discomfort during the postoperative period.^15^

Therefore, the present study was designed to compare SR (osteostatin) mixed with Gengigel and MSHA bone graft material mixed with Gengigel when used as an adjunct in open flap debridement in the treatment of bone loss due to periodontal disease in controlled diabetic patients (glycosylated hemoglobin [HbA1C] ≤7%).

This study was designed as a split-mouth investi­gation to facilitate the comparison of the two treatment procedures by eliminating patient-specific charac­teristics that might have an impact on the results of regenerative surgeries.^16^ The split-mouth design was considered adequate for evaluating regen­erative procedures in a recent systematic review.^17^ Wenzel et al^18^ reported no increase in bone fill between 6 and 12 months, which might support why the 6-month radiographic analysis was preferred in the present study.

Mol and Balasundaram^19^ compared the image quality between CBCT and conventional radiograph in assessing alveolar bone levels. They found that CBCT provided superior diagnostic accuracy and quantitative information on periodontal bone levels in three dimensions than conventional radiography.

In the present study, all the treatment modalities were well tolerated by the periodontal tissues of all the subjects who showed no signs of inflammation or postoperative pain. Also, no adverse reactions, such as allergies or abscesses, were observed in any patients.The present study showed a reduction in the mean PI and GI scores during the study period in all the groups compared to baseline values. The reduction in PI and GI in both groups might be attributed to SRP and ad­equate oral hygiene maintenance, in­structed to each patient. Regarding the PI and GI, at all evaluation periods, the t-test showed a statistically insignificant difference between the two treated groups, and it can be explained that the two treatments were performed in the same patient with the same oral hygiene and environment.

In the SR group, the reduction in GI was consistent with that reported by Nunes et al,^20^ who found that SR has an anti-inflammatory effect by reducing the release of cytokine tumor necrosis factor-alpha (TNF-α) and interleukin-1 beta (IL-1β).

The reduction of GI in both groups can be attributed to the addition of Gengigel to the SR and metal-substituted hydroxyapatite. Hyaluronic acid has many important physiological and biological functions, such as the regulation of inflammation, enhanced cell migration, proliferation, differentiation, angiogenesis, osteoconduction, carrier function, bacteriostatic function, and healing with less scarring. A clinical trial explored the utility of hyaluronic acid anti-inflammatory, anti-edematous, and anti-bacterial effects in treating periodontal disease. Therefore, hyaluronic acid has been employed in the treatment of gingivitis and periodontal pockets.^21^

PPD and CAL results revealed a significant reduction in both treatment modalities at 3- and 6-month evaluation periods compared to baseline. At 3- and 6-month evaluation periods, the t-test showed a significant reduction in PPD and CAL between the two treated groups. Using an image analysis program for CBCT, there was a statistically significant reduction in VBL in both groups compared to baseline values. At baseline, there was no statistically significant difference in the mean VBL between the two groups; however, at the 6-month postoperative interval, there was a statistically significant reduction in the two groups. Regarding the BG, there was a significant increase in BG in the SR group (3.4 mm bone gain) compared to the MSHA group (1.4 mm bone gain).

This result is consistent with Ammann et al,^22^ who found that rats treated with SR over two years exhibited increased trabecular and cortical bone volumes and trabecular number and thickness. These findings were contributed to increasing alkaline phosphatase activity and IGF-I. Ammann et al^22^ showed by CT measurements that SR treatment could positively influence intrinsic bone tissue quality.

In group 2, the reduction in PPD, CAL, and bone gain was consistent with a previous report by Kasaj et al^23^ on the clinical efficacy of NcHA paste in intrabony defects, indicating PPD and CAL reduction and bone gain. Schwarz et al^24^ evaluated the healing of intrabony peri-implantitis defects following the application of two types of treatment, a NcHA or a bovine-derived xenograft combined with a collagen membrane (BDX1BG). Postoperative wound healing revealed that NHA compromised the initial adhesion of the mucoperiosteal flaps in all patients.

This study was consistent with Bhardwaj et al,^25^ who evaluated the efficacy of an indigenously prepared zinc incorporated nanohydroxyapatite (ZINH) bone graft in the treatment of intrabony defects. A split-mouth study, which consisted of 11 systemically healthy subjects with 45 sites, randomly used ZINH or nano-HA alone. Statistically significant improvements in all the clinical parameters were seen in the test sites at 12 months. They concluded that ZINH bone graft could be considered a prospective bone regenerative material.

Li et al^26^ concluded that Mn-substituted hydroxyapatites exhibited a remarkably beneficial effect on bone cells, and it has been shown that a relatively high Mn^2+^ doping concentration promotes osteocalcin production. Also, Mn-substituted hydroxyapatites were able to support human osteoblast differentiation, proliferation, and metabolism activation.Hence, the adhesion of osteoblasts to Mn-substituted hydroxyapatites is a crucial step for subsequent osteoblast functions. Mn^2+^ ions increase ligand binding affinity, integrating and activating cell adhesion.

Wu et al ^27^ reported that iron (Fe^2+^) substituted HA nanoparticles were superparamagnetic and exhibited good biocompatibility. It has also been reported that iron compounds can promote the nucleation of apatites and adsorption of salivary calcium and phosphate ions, favoring minerals’ replacement.

## Conclusion

Strontium ranelate and MSHA bone graft material exhibited good results and an ability to augment the bone, reducing the pocket depth, improving clinical attachment levels, and promoting defect fill. However, it should be noted that the effect of MSHA was less than the strontium ranelate bone graft material. Cone-beam CT provides high-resolution images that can be used to gather diagnostic and quantitative information on periodontal bone health. The 3D images are ideal for evaluating the intrabony defects and assessing the treatment outcomes.

### Recommendations

Further studies are required to investigate larger numbers of patients with different types of periodontal defects and more extended periods of follow up to evaluate the efficiency of strontium ranelate bone graft and metal-substituted hydroxyapatite in improving the periodontal intrabony defects in chronic periodontitis patients.

## Authors’ contributions

KOH: This author was responsible for the study’s concept and design and critically revised the manuscript for important intellectual content. AAA: This author did the study’s clinical work, including collecting the patients and performing the surgeries. This author conducted a conception and design along with analysis and interpretation of data. EEA: This author undertook the acquisition and collection of data. Furthermore, this author revised and approved the manuscript to be published. MB: This author undertook the acquisition and collection of radiologic data.

## Competing interests

The authors declare no conflict of interests.

## Ethics approval

Ethics Committee number: 03111218.
